# Gastroprotective Activity of Violacein Isolated from *Chromobacterium violaceum* on Indomethacin-Induced Gastric Lesions in Rats: Investigation of Potential Mechanisms of Action

**DOI:** 10.1155/2014/616432

**Published:** 2014-08-05

**Authors:** Paulrayer Antonisamy, Ponnusamy Kannan, Adithan Aravinthan, Veeramuthu Duraipandiyan, Mariadhas Valan Arasu, Savarimuthu Ignacimuthu, Naif Abdullah Al-Dhabi, Jong-Hoon Kim

**Affiliations:** ^1^Biosafety Research Institute, College of Veterinary Medicine, Chonbuk National University, 664-14 1GA, Duck Jin-Dong, Deokjin-gu, Jeonju City, Jeollabuk-do 561-756, Republic of Korea; ^2^Division of Ethnopharmacology, Entomology Research Institute, Loyola College, Chennai, Tamil Nadu 600 034, India; ^3^Department of Botany and Microbiology, Addiriyah Chair for Environmental Studies, College of Science, King Saud University, P.O. Box 2455, Riyadh 11451, Saudi Arabia

## Abstract

*Chromobacterium violaceum*, Gram-negative bacteria species found in tropical regions of the world, produces a distinct deep violet-colored pigment called violacein. In the present study, we investigated whether violacein can promote a gastroprotective effect and verified the possible mechanisms involved in this action. For this study, an indomethacin-induced gastric ulcer rat model was used. The roles of biomolecules such as MPO, PGE_2_, pro- and anti-inflammatory cytokines, growth factors, caspase-3, NO, K^+^ATP channels, and *α*_2_-receptors were investigated. Violacein exhibited significant gastroprotective effect against indomethacin-induced lesions, while pretreatment with L-NAME and glibenclamide (but not with NEM or yohimbine) was able to reverse this action. Pretreatment with violacein also restored cNOS level to normal and led to attenuation of enhanced apoptosis and gastric microvascular permeability. Our results suggest that violacein provides a significant gastroprotective effect in an indomethacin-induced ulcer model through the maintenance of some vital protein molecules, and this effect appears to be mediated, at least in part, by endogenous prostaglandins, NOS, K^+^ATP channel opening, and inhibition of apoptosis and gastric microvascular permeability.

## 1. Introduction

Nonsteroidal anti-inflammatory drugs (NSAIDs) remain the first line therapy for rheumatoid arthritis and osteoarthritis. Unfortunately, their therapeutic effects are problematic due to gastrointestinal toxicity [1], primarily via inhibition of prostaglandin synthesis [2], neutrophil infiltration [3], nitric oxide imbalance [4], induction of apoptosis [5], and production of free radicals [6]. These radicals play a prominent role in microvascular injury [7] and cell death [5].

In the pharmaceutical industry, microorganisms have become an important source of natural products. Nearly 63% of commercially available drugs are directly or indirectly derived from microorganisms, plants, or animals [8].* Chromobacterium violaceum *is a Gram-negative *β*-proteobacterium, facultative anaerobic, saprophyte, free-living soil- and water-associated microorganism found in water body soils in tropical and subtropical regions of the world. Violacein, a purple pigment, is a main characteristic of this bacterium and exhibits significant activity against essential tropical pathogens such as* Mycobacterium tuberculosis*,* Trypanosoma cruzi*,* Leishmania *sp., and* Plasmodium falciparum*. It is reported to have antifungal, antioxidant, antitumor, bactericidal, cytotoxic, and antiviral activities [9]. Several studies have shown that violacein is also capable of inducing apoptosis in a variety of cancer cell lines, including leukemia lineages, suggesting a promising clinical application in cancer treatment. The therapeutic application of violacein to cancer chemoprevention has been the focus of recent studies [10, 11]. Violacein has been shown to induce apoptosis in HL60 leukemic cells but is ineffective in normal human lymphocytes and monocytes [12]. In our previous studies, we showed that violacein possesses immunomodulatory, analgesic, antipyretic, antidiarrheal, and antiulcerogenic activities [13, 14].

In this study, we investigated the gastroprotective effect of violacein in an indomethacin-induced ulcer model in Wister rats to determine its mechanisms of action.

## 2. Materials and Methods

### 2.1. Animals

Adult Wistar albino rats (200–220 g) of both sexes were used for experiments. Animals were housed with a 12 h light/dark cycle at 25 ± 1°C at a relative moisture of 60–70%; they had access to diet and water* ad libitum *and underwent at least two weeks of adaptation before starting the experiments. All studies were carried out using six animals in each group. All animal experiments were conducted according to the ethical norms approved by the Ministry of Social Justice and Empowerment, Government of India, and the procedures of the Institutional Animal Ethics Committee.

### 2.2. Chemicals and Drugs

**Figure 1:**
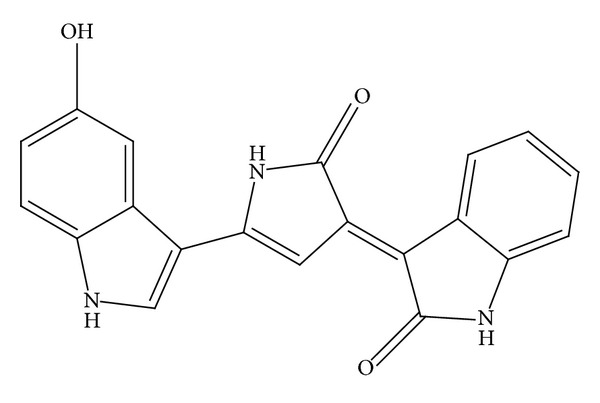
Structure of violacein.

Indomethacin, omeprazole, celecoxib, SC560, N-G-nitro-L-arginine methyl ester (L-NAME), N-ethylmaleimide (NEM), yohimbine, and glibenclamide were obtained from Sigma-Aldrich (Sigma Chemicals Co., St. Louis, MO, USA). Carboxymethyl cellulose (CMC) was obtained from Himedia (Mumbai, India). The PGE_2_ EIA kit, vascular endothelial growth factor (VEGF), hepatocyte growth factor (HGF), epidermal growth factor (EGF), and enzyme-linked immunosorbent assay (ELISA) kits were purchased from Cayman Chemical (Ann Arbor, MI, USA). Tumor necrosis factor (TNF)-*α*, interleukin (IL)-1*β*, IL-4, IL-6, and IL-10 ELISA kits were purchased from Pierce Biotechnology (Rockford, IL, USA). The apoptosis assay kit was acquired from Boehringer Mannheim, and caspase-3 activity assays were conducted using the Quanti Zyme assay system from Biomol Research Laboratories, Inc. (USA). All other chemicals used were of analytical reagent grade. The test compound, violacein, was isolated from* C. violaceum* ESBV 4400, which was isolated from forest water body soil samples from Kolli Hills of Tamil Nadu, India, at latitude 11° 10′ to 11° 30′ N and longitude 78° 15′ to 78° 30′ E (Figure 1) and reported in our previous study [14].

### 2.3. Determination of Doses

To determine the lowest effective dose of violacein, gastric ulcers were induced by indomethacin followed by treatment with violacein. After fasting for 24 h, rats were distributed into eight groups (*n* = 6/group). Animals orally received vehicle alone (0.5 mL of 0.5% CMC) for* sham* and indomethacin treated control group, omeprazole as the positive control (40 mg/kg), or violacein (10, 20, 40, 80, or 160 mg/kg). After 30 min, the animals orally received 20 mg/kg of indomethacin except sham treated group. Six hours later, the animals were sacrificed under ether anesthesia, and the stomach was surgically removed, immersed in 5% formalin for 30 min, and opened along the greater curvature to macroscopically examine lesions according to the ulcer score described by previous method [15].

### 2.4. Gastric Damage Induced by Indomethacin

Rats (*n* = 6) were treated with* sham* (0.5 mL of 0.5% CMC), vehicle + Indo (0.5 mL of 0.5% CMC), violacein (40 mg/kg, p.o.), omeprazole (40 mg/kg p.o.), SC560 + violacein (5 mg/kg p.o. + 40 mg/kg p.o.), celecoxib + violacein (3.5 mg/kg p.o. + 40 mg/kg p.o.), L-NAME + violacein (50 mg/kg i.p. + 40 mg/kg p.o.), NEM + violacein (10 mg/kg s.c. + 40 mg/kg p.o.), yohimbine + violacein (2 mg/kg i.p. + 40 mg/kg p.o.), or glibenclamide + violacein (5 mg/kg p.o. + 40 mg/kg p.o.). All drugs were administered using 0.5% CMC as the vehicle solution. After 30 min, each group of animals except the* sham* treated group received a 20 mg/kg oral dose of indomethacin. Selective COX-1 inhibitor (SC560), COX-2 inhibitor (celecoxib), nonselective nitric oxide synthase (NOS) inhibitor (L-NAME), endogenous sulfhydryl antagonist (NEM), *α*_2_-receptors antagonist (yohimbine), and K^+^ATP channels antagonist (glibenclamide) were administered to rats 30 min before violacein treatment and 1 h prior to ulcer induction by indomethacin. Six hours later, animals were sacrificed under ether anesthesia, and the stomach was surgically removed, immersed in 5% formalin for 30 min, and opened along the greater curvature to macroscopically examine lesions according to the ulcer score described by previous method [15]:

 0 = no damage;
 1 = blood at the lumen;
 2 = pin-point erosions;
 3 = one to five small erosions <2 mm;
 4 = more than five small erosions <2 mm;
 5 = one to three large erosions >2 mm;
 6 = more than three large erosions >2 mm.

The inhibition percentage was calculated using the following formula from Demirbilek et al.: [(UI nontreated − UI treated)/UI nontreated] × 100 [16].

### 2.5. Determination of MPO Activity Level

MPO activity in the gastric mucosa was determined according to the method described previously [17]. MPO activity in gastric tissues was expressed as *μ*mol/min/mg tissue.

### 2.6. Determination of Inflammatory Mediators and Tissue Growth Factors Levels

PGE_2_, TNF-*α*, IL-1*β*, IL-4, IL-6, IL-10, VEGF, EGF, and HGF were quantified in the stomach homogenate using enzyme-linked immunosorbent assay kits according to the manufacturer's specifications. The results were expressed as pg/gm tissue or ng/gm tissue.

### 2.7. Determination of NOS Activity

Gastric mucosal NOS activity was measured spectrophotometrically with the oxidation of oxyhemoglobin to methemoglobin by NO as previously described [18, 19]. The absorption deference between 401 and 421 nm was constantly observed with a dual wavelength recording spectrophotometer at 37°C. Induced NOS (iNOS) activity was calculated by subtraction of cNOS activity from total NOS activity.

### 2.8. Determination of Apoptosis and Caspase-3 Activity Level

Apoptosis and caspase-3 levels have been assayed spectrophotometrically by previous methods [20, 21]. Apoptosis and caspase-3 levels were expressed as unit/mg protein and as pmol/mg protein, respectively.

### 2.9. Determination of Microvascular Permeability

Rats were divided into six groups, each containing six animals. Animals were fasted for 24 h prior to experiments and allowed free access to water. The first group of rats received 0.5 mL of 0.5% CMC and served as* sham* treated group. The second group was subjected to gastric injury by intragastric installation of indomethacin at a dose of 20 mg/kg and was used as the ulcer-induced group. The remaining four groups were given violacein (40 mg/kg), sucralfate (400 mg/kg), SC560 + violacein (30 mg/kg + 40 mg/kg), or celecoxib + violacein (30 mg/kg + 40 mg/kg) by intragastric administration at 1 hr before ulcer induction using indomethacin. All drugs, including indomethacin, violacein, sucralfate, SC560, and celecoxib, were suspended in 0.5% CMC. Gastric microvascular permeability was evaluated 4 h after indomethacin treatment by measuring the extravasated amount of Evan's blue dye in the mucosa according to the previously mentioned method [22]. In each animal, 1 mL of 1% (w/v) Evan's blue in sterile saline was injected intravenously 30 min before sacrifice. Under ether anesthesia, animals were sacrificed by bleeding from the descending aorta, the stomachs were removed, and the gastric mucosa was scraped off and immersed in distilled water. The dye was extracted with formamide and quantified spectrophotometrically at 620 nm, and results are expressed as *μ*g/mg protein.

### 2.10. Statistical Analysis

Data were statistically analyzed using analysis of variance (ANOVA), followed by Student's* t*-test. A probability level lower than 0.05 was considered statistically significant.

## 3. Results and Discussion

**Figure 2:**
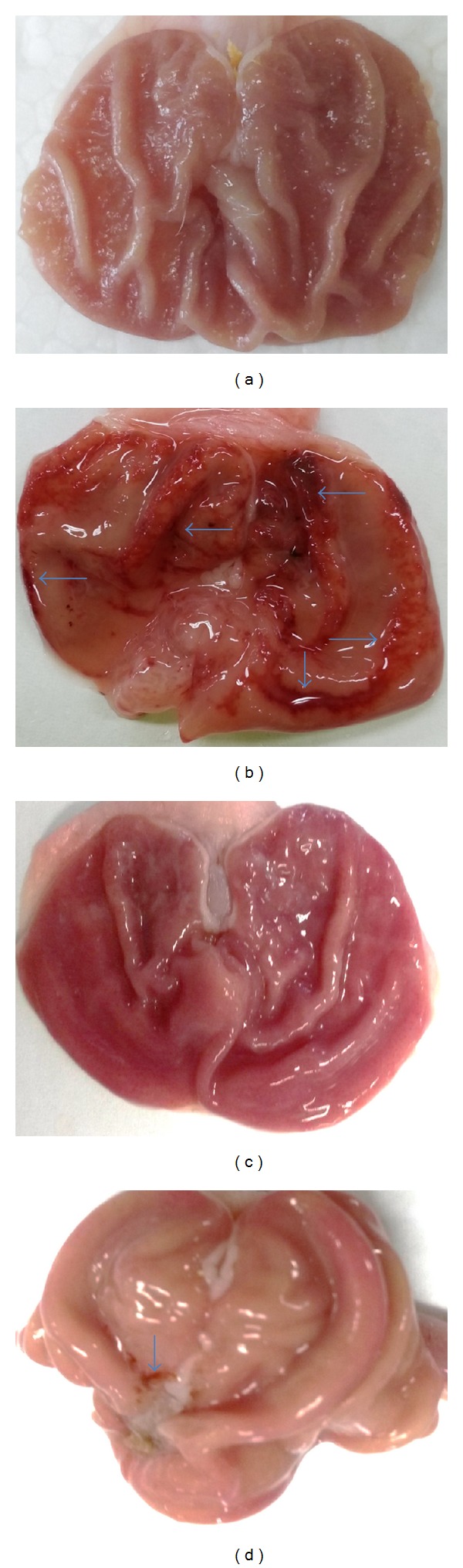
Gastroprotective activity of violacein (40 mg/kg) on indomethacin-induced gastric injury in rats. (a) Sham treated rats, (b) vehicle + indomethacin treated rats, (c) violacein (40 mg/kg) pretreated rats, and (d) omeprazole (40 mg/kg) pretreated rats. Note that indomethacin induced sever injuries to the gastric mucosa that appear as elongated bands of hemorrhage (blue arrow).

**Figure 3:**
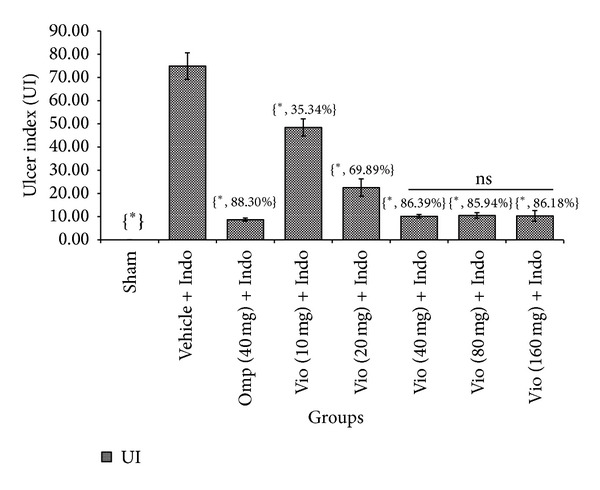
Effect of violacein (10, 20, 40, 80, and 160 mg/kg, orally) on indomethacin-induced ulcer index in rats. Values are mean ± SD (*n* = 6). _ _*P < 0.05 compare vehicle + Indo with all the groups. Values in the braces indicate ulcer index inhibition percentage. Indo: indomethacin; Vio: violacein; UI: ulcer index; ns: nonsignificant.

Because of their anti-inflammatory, analgesic, and antipyretic effects, NSAIDs are commonly used to treat rheumatoid arthritis, pyrexia, bone pain, headache, migraine, acute gout, and many other conditions [23]. High dosage, inopportune consumption, or sustained use of NSAIDs occasionally causes severe intestinal ulcer and gastroduodenal sicknesses [23]. The gastroprotective effect of violacein against NSAID-induced ulcer has not been reported until now. In the present study, we evaluated the gastroprotective effects of orally administered violacein on indomethacin-induced gastric damage in rats. Our macroscopic analyses exposed that administration of indomethacin (20 mg/kg) produced noticeable mucosal injury in the abdomen. Violacein pretreated group (Figure 2(c)) or omeprazole group (Figure 2(d)) considerably reduced gastric lesion compared to the ulcer control group, where indomethacin induced intense gastric mucosal damage in the form of elongated band of hemorrhages (Figure 2(b)). Normal group shows intact stomach without any incisions (Figure 2(a)). Regarding effective dose evaluation, the vehicle + Indo group and the 10 and 20 mg/kg groups and the 40 mg/kg dose showed a significant ulcer protective effect (*P* < 0.05). The 80 and 160 mg/kg doses of violacein produced the same effect as the 40 mg/kg dose, so 40 mg/kg was selected as the upper limit for further experiments. Rats receiving only vehicle (sham treated) showed no gastric mucosal lesions, while indomethacin administration produced mucosal lesions in rat stomachs. Compared with rats in untreated group, the indomethacin damage scores in violacein (40 mg/kg)—and omeprazole—treated groups were reduced by 86.39% and 88.30%, correspondingly (Figure 3).

**Figure 4:**
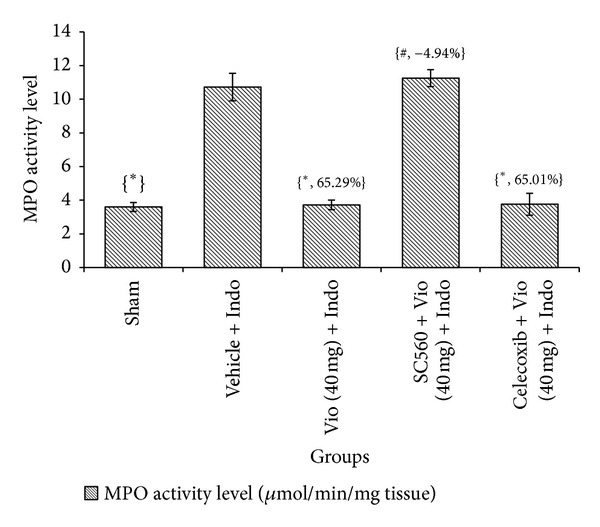
Effect of COX-1 and COX-2 inhibitors and violacein (40 mg/kg, orally) on MPO level in indomethacin-induced ulcer. Values are mean ± SD (*n* = 6). _ _*P < 0.05 compare vehicle + Indo with all the groups; _ _^#^ *P* < 0.05 compare Vio (40 mg) + Indo with SC560 (or) Celecoxib + Vio (40 mg) + Indo. Indo: indomethacin; Vio: violacein; MPO: myeloperoxidase.

**Figure 5:**
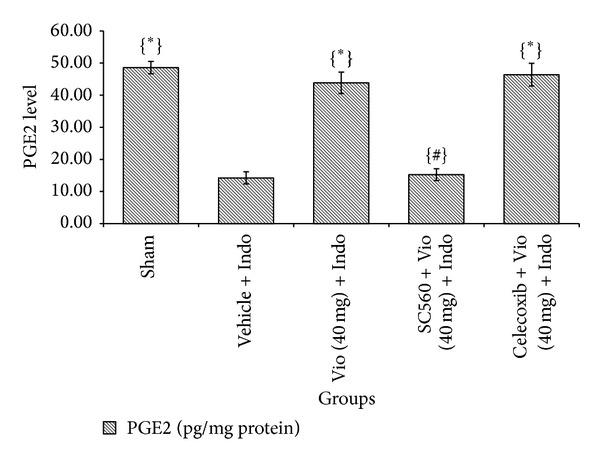
Effect of violacein (40 mg/kg, orally) on mucosal PGE_2_ level in indomethacin-induced ulcer. Values are mean ± SD (*n* = 6). _ _*P < 0.05 compare vehicle + Indo with all the groups; _ _^#^ *P* < 0.05 compare Vio (40 mg) + Indo with SC560 (or) Celecoxib + Vio (40 mg) + Indo. Indo: indomethacin; Vio: violacein.

MPO activity is known to increase in ulcerated situations and to be reduced through the curing process. MPO activity level is regularly used as a threat indicator and investigative device for evaluating the harshness of an intestinal ulcer [24]. In this study, we found that gastric MPO activity was significantly increased in the indomethacin group from 3.60 *μ*mol/min/mg tissue (sham treated) to 10.72 *μ*mol/min/mg tissue. The effect of violacein (40 mg/kg) against MPO level (3.72 *μ*mol/min/mg tissue) was greater than that of omeprazole (3.91 *μ*mol/min/mg tissue), but the difference was not statistically significant (Figure 4). NSAIDs exert their therapeutic action by inhibiting the COXs isoenzymes (COX-1 and COX-2) and reducing the levels of circulating PGs. However, the reduced levels of PGs in the intestinal mucosa are known to cause gastric ulceration and to exacerbate preexisting gastric ulcers in both rodents and humans [25]. PGs stimulate mucus and bicarbonate secretion as well as mucosal blood flow and encourage angiogenesis [24]. All of these elements contribute to reducing healing time and repairing ulcers. In this study, mucosal PGE_2_ levels were markedly suppressed (3.41-fold) in indomethacin-induced rats (*P* < 0.05) compared with sham treated group. Oral treatment with violacein and omeprazole upregulated the mucosal PGE_2_ level by 3.07- and 3.24-fold, respectively (Figure 5). Pretreatment with SC560 resulted in a significant reduction in PGE_2_ level in violacein-pretreated ulcerated rats. Thus, it is promising that violacein exerts its gastroprotective effect by stimulating synthesis of COX-1-derived PGE_2_. On the other hand celecoxib (COX-2 inhibitor) is unable to prevent the gastroprotective effect of violacein, which indicates that the COX-2 mediated prostaglandin synthesis was not involved in violacein activity. The present study also confirmed that the selective COX-1 inhibitor SC560 did not induce any injury on stomachs of normal rats but decreases the PGE_2_ level significantly (data not shown) similar to indomethacin; this is consistent with previous observations [26, 27]. However, pretreatment of SC560 completely inhibit the gastroprotective activity of violacein against indomethacin-induced ulcer; this observation clearly elucidated that the gastroprotective activity of violacein also was facilitated through the COX-1 mediated pathways. Previous reports show that PGE_2_ acts as a potential inhibitor of TNF-*α* [28, 29], apoptosis [30], and activator of K^+^ATP channels [31]; it is possible that violacein may be able to stop ulcer induction processes via the inhibition of TNF-*α*, apoptosis, and activation of K^+^ATP channels through significant production of PGE_2_ by COX-1 mediated pathways.

**Figure 6:**
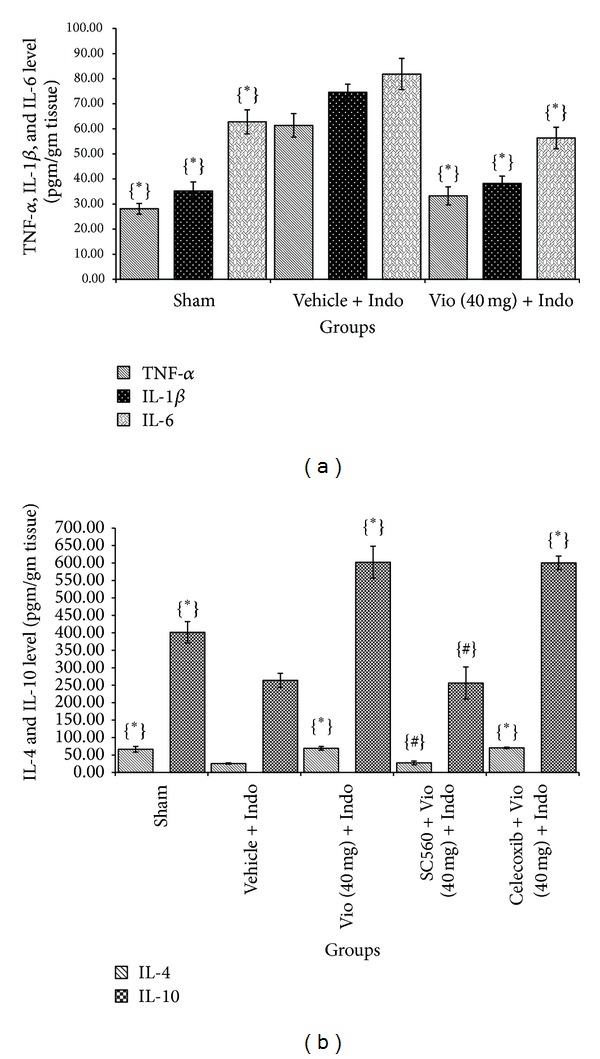
(a) Effect of violacein (40 mg/kg, orally) on proinflammatory cytokines (TNF-*α*, IL-1*β*, and IL-6) level; (b) anti-inflammatory cytokines (IL-4 and IL-10) level in indomethacin-induced ulcer. Values are mean ± SD (*n* = 6). _ _*P < 0.05 compare vehicle + Indo with all the groups; _ _^#^ *P* < 0.05 compare Vio (40 mg) + Indo with SC560 (or) Celecoxib + Vio (40 mg) + Indo. Indo: indomethacin; Vio: violacein.

**Figure 7:**
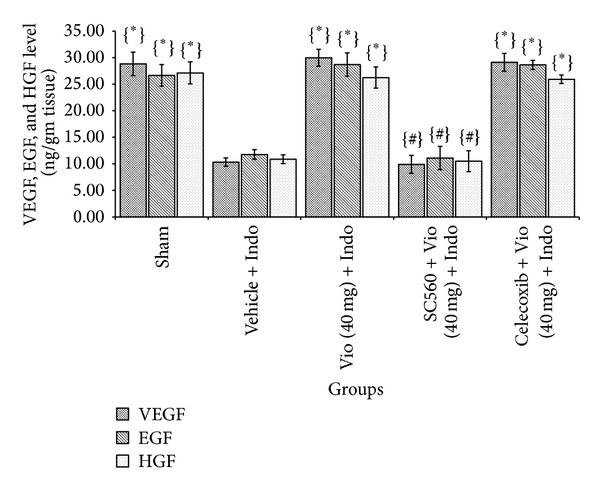
Effect of COX-1 and COX-2 inhibitors and violacein (40 mg/kg, orally) on VEGF, EGF, and HGF levels in indomethacin-induced ulcerated rats. Values are mean ± SD (*n *= 6). _ _*P < 0.05 compare vehicle + Indo with all the groups; _ _^#^ *P* < 0.05 compare Vio (40 mg) + Indo with SC560 or Celecoxib + Vio (40 mg) + Indo. Indo: indomethacin; Vio: violacein.

Indomethacin-induced ulcer shows increased expression of proinflammatory cytokines [32], which are associated with the degree of ulceration. Indomethacin administration elevated proinflammatory Th1 cytokines and reduced anti-inflammatory cytokines [33]. ELISA study illustrated that indomethacin stimulated TNF-*α* (2.18-fold), IL-1*β* (2.11-fold), and IL-6 (1.30-fold) and downregulated IL-4 (2.59-fold), IL-10 (1.52-fold), VEGF (2.79-fold), EGF (2.26-fold), and HGF (2.49-fold) levels. Violacein at a dose of 40 mg/kg significantly (*P* < 0.05) reduced proinflammatory cytokine (TNF-*α*, IL-1*β*, and IL-6) level (1.84-fold, 1.95-fold, and 1.45-fold, resp.) (Figure 6(a)) and increased anti-inflammatory cytokines (IL-4 and IL-10) level (2.69-fold and 2.28-fold, resp.) (Figure 6(b)) and growth factors (VEGF, EGF, and HGF) level (2.90- fold, 2.43-fold, and 2.41-fold, resp.) compared with the indomethacin-induced ulcerated untreated group (Figure 7). Overall above results explicate that the violacein treatment reduced proinflammatory cytokines (TNF-*α*, IL-1*β*, and IL-6) and concurrently increased the levels of tissue IL-4 and IL-10, all of which may contribute to its healing effect.

TNF-*α* seems to be a crucial contributor to many forms of intestinal mucosal injury, including that during the application of NSAIDs. NSAIDs have been shown to markedly elevate the level of TNF-*α*. Inhibition of TNF-*α* production results in attenuation of the harmful effects of NSAIDs in the rat intestine [34]. Prostaglandins are potent inhibitors of TNF-*α* release from both macrophages [29] and mast cells [28]. IL-10 has a central role in the downregulation of the inflammatory cascade by depressing the production of a number of proinflammatory cytokines [35] and enhancing the production of anti-inflammatory cytokines [36]. In this study violacein enhances IL-10 and inhibited TNF-*α* significantly, which clearly indicates the ulcer curing ability of violacein on NSAID induced ulcer. VEGF is a growth factor that increases ulcer healing by stimulating angiogenesis [37]. Likewise, HGF aids angiogenesis, through multiple mechanisms including COX activation, and increases EGF expression that eventually accelerates gastroduodenal ulcer healing by increasing gastric mucin and diminishing gastric acid secretion [38]. We determined that indomethacin administration significantly decreased mucosal VEGF, EGF, and HGF levels compared with the vehicle-treated control group, but violacein treatment considerably enriched growth factors levels. After COX-1 inhibitor pretreatment, ulcer index (UI) and MPO level were significantly increased followed by reduction of tissue VEGF, EGF, HGF, IL-4, IL-10, and PGE_2_ levels. However, the COX-2 inhibitor celecoxib did not affect the therapeutic activity of violacein at any level (*P* < 0.05).

**Figure 8:**
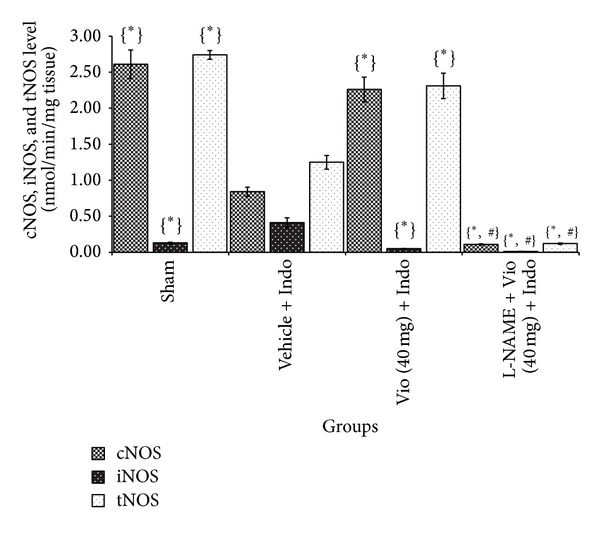
Effect of L-NAME (NOS inhibitor) and violacein (40 mg/kg, orally) on cNOS, tNOS, and iNOS levels in indomethacin-induced ulcerated rats. Values are mean ± SD (*n *= 6). _ _*P < 0.05 compare vehicle + Indo with all the groups; _ _^#^ *P* < 0.05 compare Vio (40 mg) + Indo with L-NAME + Vio (40 mg) + Indo. Indo: indomethacin; Vio: violacein; L-NAME: N-G- nitro- L-arginine methyl ester.

Gastric mucosal cNOS activity was significantly decreased in the indomethacin-induced ulcer group. In contrast, the iNOS activity in the gastric mucosa of rats subjected to indomethacin induction was significantly increased [39]. Treatment with violacein significantly increased cNOS and decreased iNOS, but this activity was inhibited by L-NAME, a nonspecific inhibitor, which confirms the involvement of NOS in violacein-mediated gastroprotection on indomethacin-induced ulcers (Figure 8).

**Figure 9:**
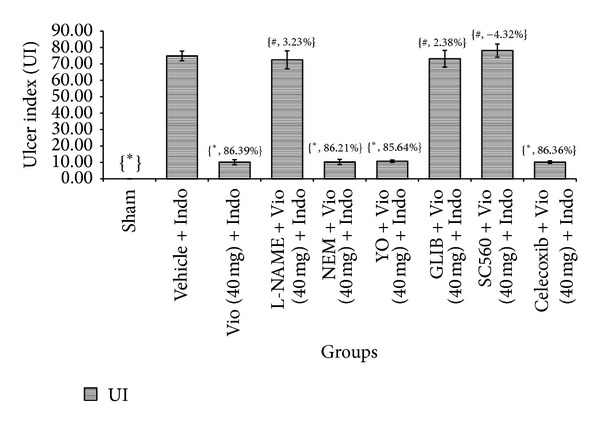
Effect of L-NAME (NOS inhibitor), NEM (NP-SH antagonist), yohimbine (*α*_2_-receptors antagonist), glibenclamide (K^+^ATP-channels antagonist), SC560 (COX-1 inhibitor), and Celecoxib (COX-2 inhibitor) on gastroprotective activity of violacein against indomethacin-induced ulcer. Values are mean ± SD (*n *= 6). _ _*P < 0.05 compare vehicle + Indo with all the groups; _ _^#^ *P* < 0.05 compare Vio (40 mg) + Indo with L-NAME, NEM, YO, GLIB, SC560, or Celecoxib + Vio (40 mg) + Indo. Values in the braces indicate ulcer index inhibition percentage. Indo: indomethacin; Vio: violacein; L-NAME: N-G-nitro-L-arginine methyl ester; NEM:* N*-ethylmaleimide; YO: yohimbine; GLIB: glibenclamide.

Nonprotein endogenous sulfhydryl (NP-SH) compounds are important for the maintenance of gastric mucosal integrity [40]. These SH groups have the ability to bind to the free radicals generated by noxious agents, thus controlling the production and nature of mucus [40]. However, our results showed significant attenuations in the gastric ulcer area after the blockage of NP-SH compounds by NEM in groups treated with violacein (40 mg/kg) in comparison with the indomethacin-induced ulcer group, suggesting that the gastroprotective effects of violacein are not involved in maintenance of NP-SH compounds (Figure 9). The opening of K^+^ATP channels, a class of ligand-gated proteins, appears to be involved with a variety of physiologic functions of the stomach, such as gastric blood flow regulation, acid secretion, and stomach contractility [41]. In fact, Peskar et al. demonstrated that endogenous prostaglandins act as activators of K^+^ATP channels, and this mechanism, at least in part, mediates gastroprotection [42]. A previous study by Peskar et al. suggested the participation of K^+^ATP channels in an indomethacin-induced ulcer model in which prostaglandins were shown as probable activators of these channels [42]. In this way, our results showed that the gastroprotection mechanism of violacein was K^+^ATP-channel dependent, since its gastroprotective effects were reverted by pretreatment with glibenclamide, a potent antagonist of these channels (Figure 9). From these data, we suggest participation of K^+^ATP channels in the gastroprotective effects of violacein, in which prostaglandins could be involved in the activation of these channels. Presynaptic *α*_2_-receptors mediate several responses in the gastrointestinal tract and are involved in the regulation of gastric acid secretion [43]. Pretreatment with the *α*_2_-receptor antagonist yohimbine failed to effectively block the gastroprotective effect of violacein (40 mg/kg) against indomethacin-induced ulcers (Figure 9), suggesting that the gastroprotective effect of violacein is not mediated by *α*_2_-receptors. L-NAME significantly inhibited the gastroprotection produced by violacein (Figure 9), suggesting that NO participates in gastroprotection. It is well known that NO is involved in the modulation of gastric mucosal integrity and in the regulation of acid and alkaline secretion, mucus secretion, and gastric mucosal blood flow [44].

**Figure 10:**
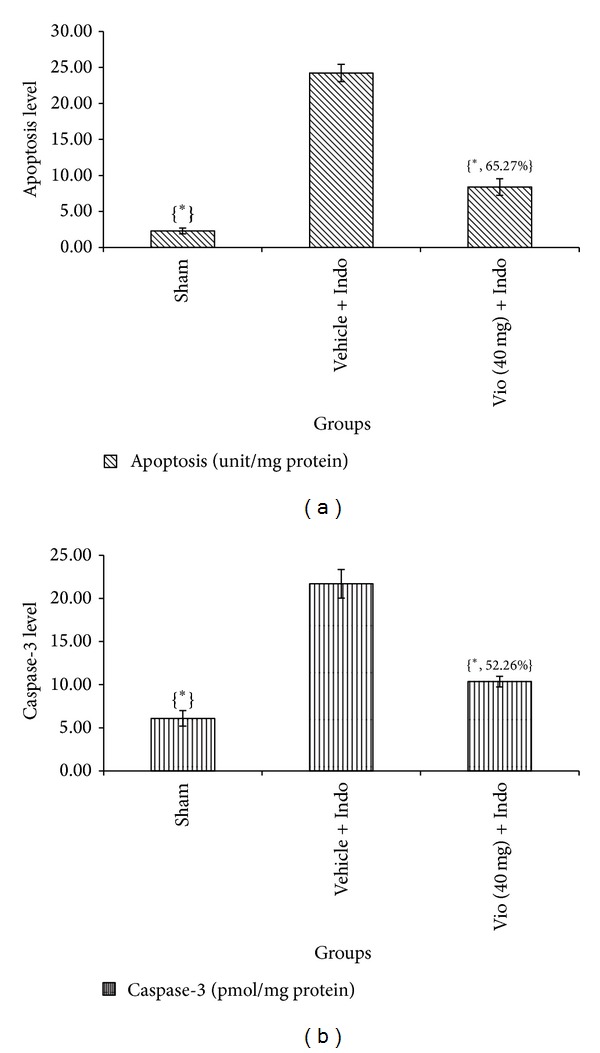
(a) Effect of violacein (40 mg/kg, orally) on apoptosis level; (b) caspase-3 level in indomethacin-induced ulcerated rats. Values are mean ± SD (*n *= 6). _ _*P < 0.05 compare Vehicle + Indo with all the groups. Values in the braces indicate apoptosis and caspase-3 level inhibition percentage. Indo: indomethacin; Vio: violacein.

**Figure 11:**
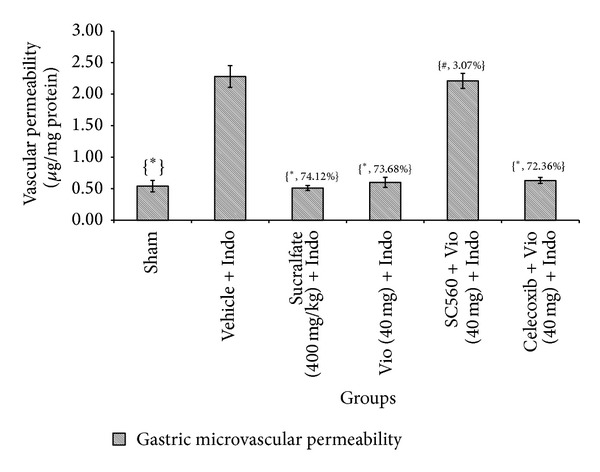
Effect of COX-1 and COX-2 inhibitors and violacein (40 mg/kg, orally) on gastric microvascular permeability induced by indomethacin in rats. Values are mean ± SD (*n *= 6). _ _*P < 0.05 compare vehicle + Indo with all the groups; _ _^#^ *P* < 0.05 compare Vio (40 mg) + Indo with SC560 or Celecoxib + Vio (40 mg) + Indo. Indo: indomethacin; Vio: violacein.

Enhancement of caspase-3 activation and considerable epithelial cell apoptosis are important pathological events during NSAIDs-induced cytotoxicity [33]. The amplification and propagation of the cell death signaling cascade induced by TNF-*α* involve the activation of a family of specific cysteine proteases known as caspases, which are under the regulatory control of nitric oxide [45]. Indomethacin displays its effects on apoptogenic signal propagation through the induction of TNF-*α* [46]; our present experiments showed that violacein significantly inhibited TNF-*α*. The apoptotic index in the indomethacin-induced group was 10.48-fold higher than that in the normal control group. Pretreatment with violacein caused a 65.27% reduction in DNA fragmentation (Figure 10(a)). The caspase-3 activity in the indomethacin-induced group was 3.55-fold higher than in the sham treated group. Pretreatment with violacein caused a 52.26% reduction in caspase-3 activity (Figure 10(b)). Based on these results, it is possible that violacein may able to inhibit these apoptogenic processes by the inhibition of TNF-*α*. The effects of violacein, sucralfate, SC560, and celecoxib on the indomethacin-induced microvascular permeability of rat stomach are depicted in Figure 11. Indomethacin increased gastric microvascular permeability by 4.2-fold. Both violacein and sucralfate ameliorated indomethacin-induced gastric microvascular permeability (73.68% and 74.12%, resp.). Microvascular permeability was significantly increased after COX-1 inhibitor pretreatment along with violacein; however, the COX-2 inhibitor celecoxib did not affect the therapeutic activity of violacein (40 mg/kg). The above result indicates the involvement of COX-1 in the process of inhibition of vascular permeability. These results are consistent with a previous report [27].

The results of our present study demonstrate that violacein presented significant gastroprotective effects in an indomethacin-induced gastric ulcer model and that appear to be mediated, at least in part, by endogenous prostaglandins, nitric oxide, K^+^ATP channel opening, upregulation of the levels of mucosal growth factors, maintenance of the balance of pro- and anti-inflammatory cytokines, antiapoptotic function, and attenuation of enhanced gastric microvascular permeability. These findings indicate that violacein may be a useful natural gastroprotective tool. However, further studies are required to evaluate the exact mechanism involved in its action as well as to better investigate the safety profile of violacein use.
